# Supplementary material for: Estimating total human deaths from rabies: A statistical framework leveraging reports of rabies transmission from deceased organ and tissue donors
Source: PLoS Negl Trop Dis. 2026 Sep 21;20(9):e0014731. doi: 10.1371/journal.pntd.0014731 (PMC13592719; doi:10.1371/journal.pntd.0014731)
Supplement: Supplementary file 6 [file pntd.0014731.s005.zip › Result_A/Switzerland/Figure_S1_Switzerland.pdf]

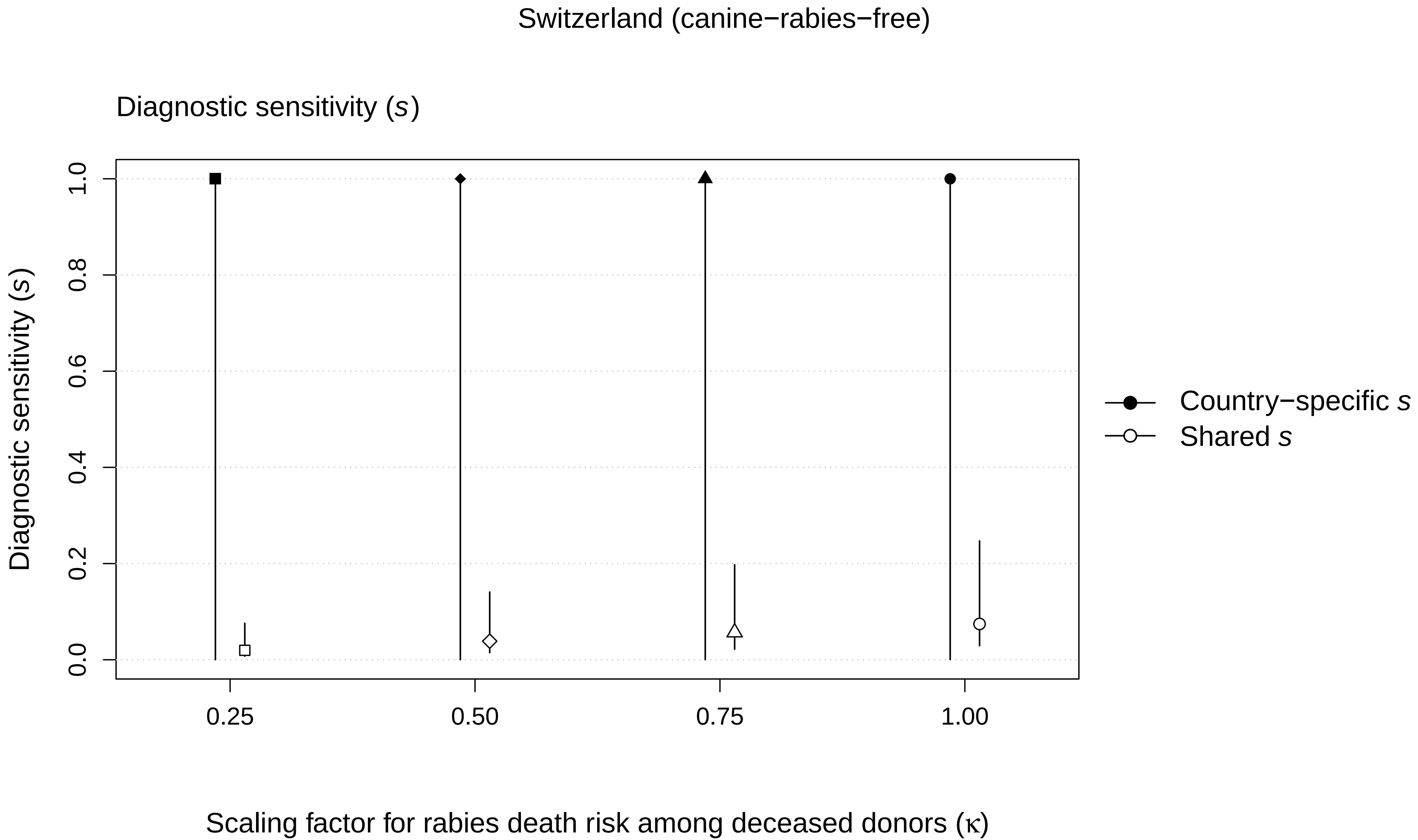
Switzerland (canine−rabies−free)
Diagnostic sensitivity (s)
Diagnostic sensitivity (s)
0.0
0.2
0.4
0.6
0.8
1.0
0.25
0.50
0.75
1.00
Scaling factor for rabies death risk among deceased donors (κ)
Country−specific s
Shared s
